# Prognostic value of normal cardiac MRI in patients with suspected arrhythmogenic right ventricular dysplasia

**DOI:** 10.1186/1532-429X-11-S1-P56

**Published:** 2009-01-28

**Authors:** Mario Njeim, Gurjit Singh, Leonidas Tzogias, Claudio Schuger, Milan V Pantelic, Khalid Nour, Mouaz H Al-Mallah

**Affiliations:** Henry Ford Hospital, Detroit, MI USA

**Keywords:** Cardiomyopathy, Cardiac Magnetic Resonance, Right Ventricle, Cardiac Magnetic Resonance Imaging, Supraventricular Tachycardia

## Background

Arrhythmogenic right ventricular dysplasia (ARVD) is an inherited cardiomyopathy characterized by ventricular arrhythmias and structural abnormalities of the right ventricle (RV). The aim of this study is to investigate the prognostic value of normal cardiac magnetic resonance imaging (CMR) in patients with suspected ARVD.

## Methods

We included patients with clinically suspected ARVD who underwent CMR between 1/1/2003 and 12/31/2007. Imaging protocol included steady-state acquisition cine imaging, T(1)-weighted black blood imaging with and without fat suppression and post-contrast delayed enhancement on a 1.5-T scanner to evaluate ventricular function and morphology, fatty infiltration and regional myocardial fibrosis. Patient who had CMR major criteria for ARVD were excluded from this analysis. In addition, patients in whom the clinical workup revealed alternative diagnosis were excluded. Included patients were followed up till 9/2008 for the incidence of all cause mortality, re-hospitalization for arrhythmic causes and (implantable defibrillator) ICD implantation.

## Results

A total of 66 patients (mean age 44.4 years, 56% males) met the inclusion criteria. After a mean follow-up duration of 3.2 years, no patients died. None of the included patients developed ARVD or arrhythmogenic right ventricular cardiomyopathy. However, 12 (18%) patients were re-hospitalized for arrhythmic causes including 4 patients with supraventricular tachycardia and 4 patients with right ventricular outflow tachycardia, three of which required ICD implantation.

## Conclusion

In patients with suspected ARVD, a normal CMR has a high negative predictive value for the development of death or future ARVD. However, an alternative diagnosis may be found in up to 18% of these patients on follow-up. Serial reevaluation for other arrhythmic causes may be warranted in symptomatic patients.

